# *ABCA4* Gene Screening in a Chinese Cohort With Stargardt Disease: Identification of 37 Novel Variants

**DOI:** 10.3389/fgene.2019.00773

**Published:** 2019-09-05

**Authors:** Fang-Yuan Hu, Jian-kang Li, Feng-Juan Gao, Yu-He Qi, Ping Xu, Yong-Jin Zhang, Dan-Dan Wang, Lu-Sheng Wang, Wei Li, Min Wang, Fang Chen, Si-Mai Shen, Ge-Zhi Xu, Sheng-Hai Zhang, Qing Chang, Ji-Hong Wu

**Affiliations:** ^1^Eye Institute, Eye and ENT Hospital, College of Medicine, Fudan University, Shanghai, China; ^2^State Key Laboratory of Medical Neurobiology, Institutes of Brain Science and Collaborative Innovation Center for Brain Science, Shanghai Medical College, Fudan University, Shanghai, China; ^3^Shanghai Key Laboratory of Visual Impairment and Restoration, Science and Technology Commission of Shanghai Municipality, Shanghai, China; ^4^Key Laboratory of Myopia (Fudan University), Chinese Academy of Medical Sciences, National Health Commission, Shanghai, China; ^5^BGI-Shenzhen, Shenzhen, China; ^6^Department of Computer Science, City University of Hong Kong, Kowloon, Hong Kong; ^7^BGI Education Center, University of Chinese Academy of Sciences, Shenzhen, China; ^8^Laboratory of Genomics and Molecular Biomedicine, Department of Biology, University of Copenhagen, Copenhagen, Denmark; ^9^Shenzhen Engineering Laboratory for Birth Defects Screening, BGI-Shenzhen, Shenzhen, China; ^10^University of Sichuan, Sichuan, China

**Keywords:** STGD1, next-generation sequencing, *ABCA4* gene, mutation spectrum, variant frequency

## Abstract

**Purpose:** To clarify the mutation spectrum and frequency of *ABCA4* in a Chinese cohort with Stargardt disease (STGD1).

**Methods:** A total of 153 subjects, comprising 25 families (25 probands and their family members) and 71 sporadic cases, were recruited for the analysis of *ABCA4* variants. All probands with STGD1 underwent a comprehensive ophthalmologic examination. Overall, 792 genes involved in common inherited eye diseases were screened for variants by panel-based next-generation sequencing (NGS). Variants were filtered and analyzed to evaluate possible pathogenicity.

**Results:** The total variant detection rate of at least one *ABCA4* mutant allele was 84.3% (129/153): two or three disease-associated variants in 86 subjects (56.2%), one mutant allele in 43 subjects (28.1%), and no variants in 24 subjects (15.7%). Ninety-six variants were identified in the total cohort, which included 62 missense (64%), 15 splicing (16%), 11 frameshift (12%), 6 nonsense (6%), and 2 small insertion or deletion (2%) variants. Thirty-seven novel variants were found, including a *de novo* variant, c.4561delA. The most prevalent variant was c.101_106delCTTTAT (10.5%), followed by c.2894A > G (6.5%) and c.6563T > C (4.6%), in STGD1 patients from eastern China.

**Conclusion:** Thirty-seven novel variants were detected using panel-based NGS, including one *de novo* variant, further extending the mutation spectrum of *ABCA4*. The common variants in a population from eastern China with STGD1 were also identified.

## Introduction

Stargardt disease (STGD1, OMIM 248200), also known as juvenile macular degeneration, is a hereditary macular dystrophy characterized by bilateral or sequential central visual loss in early adolescence (North et al., 2014; Tanna et al., 2017). STGD1 is one of the most common causes of macular dystrophy in childhood, accounting for about 7% of all retinal dystrophy, with an incidence of 1:10,000 (Blacharski, 1988). The fundus of STGD1 patients commonly presents with yellow-white flecks, bull’s eye maculopathy, a beaten bronze appearance of the macula, and atrophy of retinal pigment epithelium (RPE). It is caused by the massive deposition of lipofuscin in RPE, followed by photoreceptor cell death and eventually loss of vision (Aaberg and Han, 1987; Anderson et al., 1995; Fishman et al., 1999; Lewis et al., 1999). STGD1 is an autosomal recessive disorder caused by variants in the ATP-binding cassette subfamily A member 4 (*ABCA4*) (Allikmets, 1997).

*ABCA4* (OMIM 601691), located on chromosome 1p22.1, is composed of 50 exons and specifically expressed in retina photoreceptors (Nasonkin et al., 1998). The transmembrane protein encoded by *ABCA4* is a member of the superfamily of ATP-binding cassette transporters and is mainly involved in the transport of N-retinylidene-phosphatidylethanolamine (NRPE), an intermediate metabolite of vitamin A, from the disc membrane of the photoreceptor outer segment to the cytoplasm (Molday and Zhang, 2010; Quazi et al., 2012; Quazi and Molday, 2014). Therefore, *ABCA4* promotes the removal of toxic retinal phospholipid compounds from photoreceptor cells, and its dysfunction results in the degeneration of photoreceptor cells (Molday, 2007; Molday et al., 2009). Variants in the *ABCA4* gene are responsible for STGD1 (Allikmets, 1997), retinitis pigmentosa (RP) (Cremers et al., 1998; Rozet et al., 1999), cone–rod dystrophy (CRD) (Cremers et al., 1998; Maugeri et al., 2000), and retinal dystrophy (Singh et al., 2006). It has been reported that *ABCA4* is the only gene confirmed to be associated with STGD1 (Allikmets, 1997). To date, approximately 1200 disease-related variants have been identified in retinopathy caused by *ABCA4* (Cornelis et al., 2017) (Human Gene Mutation Database, HGMD, http://www.lovd.nl/ABCA4). Among them, nearly 900 variants are associated with STGD1, and the majority of variant types are missense. The variant detection rate of at least one *ABCA4* mutant allele ranges from 70% to 90% in different STGD1 cohorts (Fujinami et al., 2013; Riveiro-Alvarez et al., 2013; Zernant et al., 2014b; Jiang et al., 2016; Smaragda et al., 2018). Although the disease-causing *ABCA4* variants are extremely heterogeneous, previous studies have confirmed frequent *ABCA4* variants with ethnic specificity, such as c.2588G > C in the Western European population (Maugeri et al., 1999), p.Y808* in Chinese patients (Jiang et al., 2016), c.5714+5G > A in a Greek cohort (Smaragda et al., 2018), p.N965S in Denmark (Rosenberg et al., 2007), p.A1773V in Mexico (Chacon-Camacho et al., 2013), p.R1129L in Spain (Riveiro-Alvarez et al., 2013), and p.R2107H in African American patients (Zernant et al., 2014a).

In this study, we investigated the spectrum of *ABCA4* variants using panel-based next-generation sequencing (NGS) technology in a Chinese cohort with STGD1. The purpose of this study is to extend the mutation spectrum of *ABCA4*. Our analysis identified 37 novel disease-associated variants of *ABCA4* and determined the prevalent *ABCA4* variants mainly in the population of eastern China. Furthermore, we identified one novel heterozygous variant, c.4561delA, as a *de novo* variant in the *ABCA4* gene in one STGD1 patient.

## Materials and Methods

### Subjects and Ethics Statement

A total of 153 subjects, comprising 25 families (25 probands and their relatives) and 71 sporadic cases, were recruited at the Eye and ENT Hospital of Fudan University from 2016 to 2018. This study was approved by the Ethics Committee of the Eye and ENT Hospital of Fudan University and in accordance with the Code of Ethics of the World Medical Association (Declaration of Helsinki) for medical research involving human subjects. All participants were recruited after obtaining their informed consent.

### Clinical Assessment

All probands underwent a comprehensive ophthalmologic examination, including best corrected visual acuity (BCVA), slit-lamp biomicroscopy, fundus examination, fundus photograph, electroretinography (ERG), spectral domain optical coherence tomography (SD-OCT, Spectralis HRA + OCT, Heidelberg Engineering Inc., Heidelberg, Germany), and fundus autofluorescence (FAF, Spectralis HRA + OCT, Heidelberg, Germany). Moreover, other associated information was collected, including family history, age of onset, duration of disease, and subjective degree of vision loss. The clinical diagnosis of STGD1 was assessed by professional ophthalmologists.

### DNA Sample Collection

Genomic DNA was extracted from the peripheral blood of all subjects using the FlexiGene DNA Kit (Qiagen, Venlo, Netherlands), in accordance with the manufacturer’s protocol. DNA integrity was observed by 1% agarose gel electrophoresis. DNA samples were stored at −20°C before sequencing analysis.

### Targeted Exome Sequencing and Genetic Analyses

Panel-based NGS was performed on all subjects in this study. We designed the Target_Eye_792_V2 chip with exon-capture and untranslated regions (UTRs) of 792 genes involved in common inherited eye diseases (**Supplementary Table S1**), in collaboration with BGI-Shenzhen (Shenzhen, Guangdong, China). Genomic DNA was sheared into fragments, and fragments containing the coding exons, flanking intronic regions, and promoter regions were captured using the Agilent SureSelect Target Enrichment Kit (Agilent Technologies, Inc., USA). The enriched libraries were sequenced on a MGISEQ-2000 platform (BGI, Inc., Shenzhen, China) in accordance with the manufacturer’s protocols. Sequencing reads were mapped to the reference human genome (hg38) using a Burrows–Wheeler Aligner (BWA, http://bio-bwa.sourceforge.net/). All identified variants were annotated using the following four databases: 1,000 Genomes Project (http://browser.1000genomes.org/), dbSNP (http://www.ncbi.nlm.nih.gov/projects/SNP/), ESP6500 (http://evs.gs.washington.edu/EVS/), and ExAC (http://exac.broadinstitute.org). The variants with minor allele frequency (MAF) < 0.1% were selected to find possible deleterious variants. In addition, potential pathogenic variants were predicted using the following three online tools: Sorting Intolerant from Tolerant (SIFT, http://sift.jcvi.org/), Polymorphism Phenotyping v2 (PolyPhen-2, http://genetics.bwh.harvard.edu/pph2/), and MutationTaster software (http://www.mutationtaster.org/). Then, the remaining variants were prioritized using ClinVar (https://www.ncbi.nlm.nih.gov/clinvar/), HGMD (http://www.hgmd.cf.ac.uk/ac/index.php), and Online Mendelian Inheritance in Man (OMIM, http://www.omim.org/) in combination with their potential deleterious impacts, genotype–phenotype relationships, and variant reports. In accordance with the American College of Medical Genetics (ACMG) and genomics guidelines, variants were classified as pathogenic, likely pathogenic, uncertain significance, likely benign, or benign. Sanger sequencing was performed to confirm the candidate variants.

## Results

### Clinical Findings

**Figure 1:**
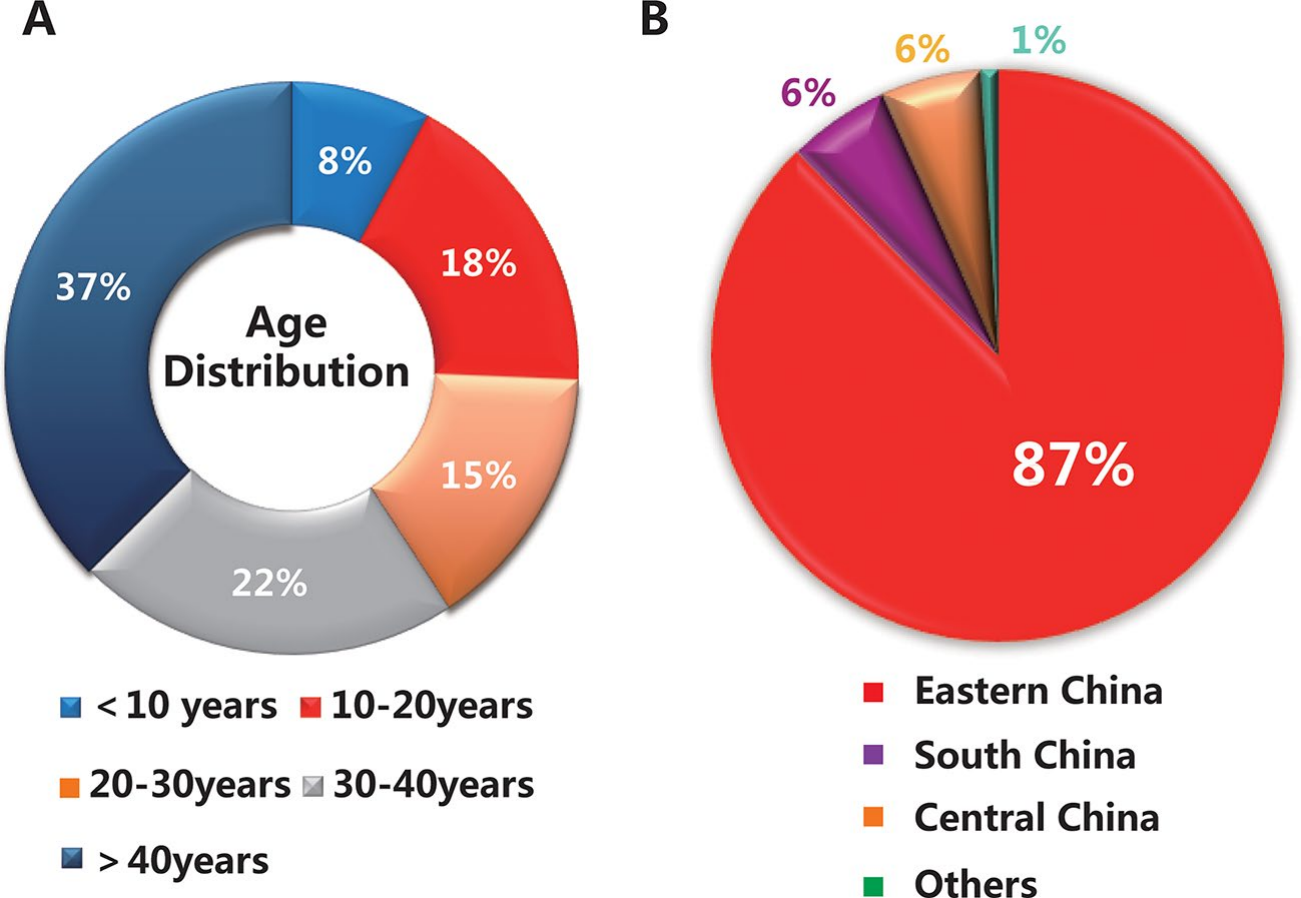
Basic information on the 153 subjects in this study. **(A)** The age distribution of the total cohort. **(B)** The geographical distribution of the population.

A total of 153 subjects were presented in this study, including 25 families (25 probands and their relatives, 82 subjects in total) and 71 sporadic cases. The average age of the total cohort was 33 years (range 5–76 years), of which the majority were under 40 years old, about 63% (95 subjects) (**Figure 1A**). As shown in **Figure 1B**, 87% of the subjects were from eastern China, while the others were from regions including south China and central China. The clinical characteristics of 101 patients with a clinically definitive diagnosis are presented in **Table 1**. The average age of the patients was 28.0 years (range 6–76 years) and the average age of onset was 19.0 years (range 2–70 years). The average duration of disease in the 101 patients was 10 years. In addition, the average BCVA of the right eye and left eye were 0.50 (range 0.1–1.0) and 0.50 (range 0.1–1.2), respectively. Fundus examination in STGD1 patients revealed macular atrophy and the degeneration of photoreceptors and RPE cells.

**Table 1:** Clinical characteristics in 101 patients with STGD1.

Average age (years)	28.0 (6–76)
Average age of onset (years)	19.0 (2–70)
Average duration of disease (years)	10.0 (1–50)
Average BCVA (right eye)	0.5 (0.1–1.0)
Average BCVA (left eye)	0.5 (0.1–1.2)

### Variant Detection Rates of *ABCA4*

Of the 153 subjects screened by panel-based NGS, *ABCA4* mutant variants were identified in 129 cases, resulting in a total variant detection rate of 84.3%: two or three *ABCA4* disease-associated variants in 86/153 (56.2%) subjects, one disease-causing allele in 43/153 (28.1%) subjects, and no variants in 24 subjects (15.7%) (**Table 2**). Furthermore, the variant detection rates in the families and sporadic cases were 87.8% and 80.3%, respectively (**Table 2**). Among 25 probands in 25 families, three *ABCA4* mutant variants were identified in only one patient, two disease-associated variants were identified in 28 patients, and no variants in one proband. In sporadic cases, potential pathogenic variants of *ABCA4* were identified in 57 of the 71 patients (80.3%). Consistent with previous studies, the majority of patients with STGD1 carried compound heterozygous variants.

**Table 2:** Variant detection rates of *ABCA4* in this study.

Variance per cases	Families (no./percentage)	Sporadic cases (no./percentage)	Total cases (no./percentage)
3	1/1.2	2/2.8	3/2.0
2	28/34.2	55/77.5	83/54.2
1	43/52.4	0/0	43/28.1
0	10/12.2	14/19.7	24/15.7
	82/100	71/100	153/100

### Genetic Analyses

**Figure 2:**
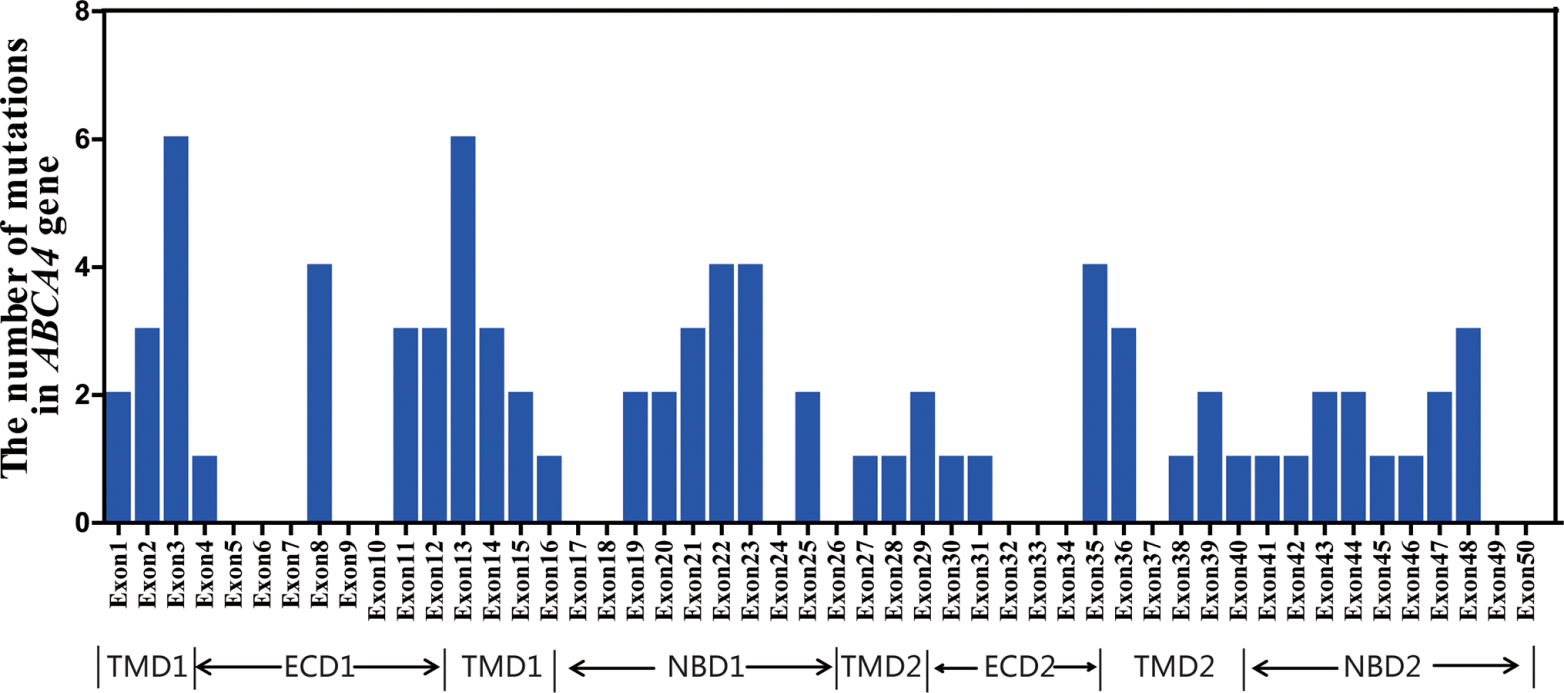
Distribution and frequency of the *ABCA4* mutant variants identified in this study.

Ninety-six distinct variants of *ABCA4* were identified in the total cohort, including 37 novel variants and 59 known variants. The detailed genetic analyses of 96 *ABCA4* variants are summarized in **Supplementary Table S2** and **Supplementary Table S3**. All variants and cases with variants are uploaded at http://www.lovd.nl/ABCA4. As shown in **Figure 2**, 96 variants were widely distributed in 50 exons of *ABCA4*. Analysis revealed six different mutant variants each in exons 3 and 13; four different variants each in exons 8, 22, 23, and 35; and one to three in each of the remaining 29 exons. In the six functional domains of the ABCA4 protein, mutant variants were mainly distributed in transmembrane domain 1 (TMD1) and nucleotide-binding domains 1 and 2 (NBD1 and NBD2). Moreover, there were 15 variants observed in the introns of *ABCA4*, including introns 7, 8, 10, 12, 13, 23, 26, 28, 29, 36, 38–40, 44, and 45. Through analysis with Human Splicing Finder (http://www.umd.be/HSF3/), these intronic mutations mainly affected splice donor or acceptor sites (**Supplementary Table S2**).

**Figure 3:**
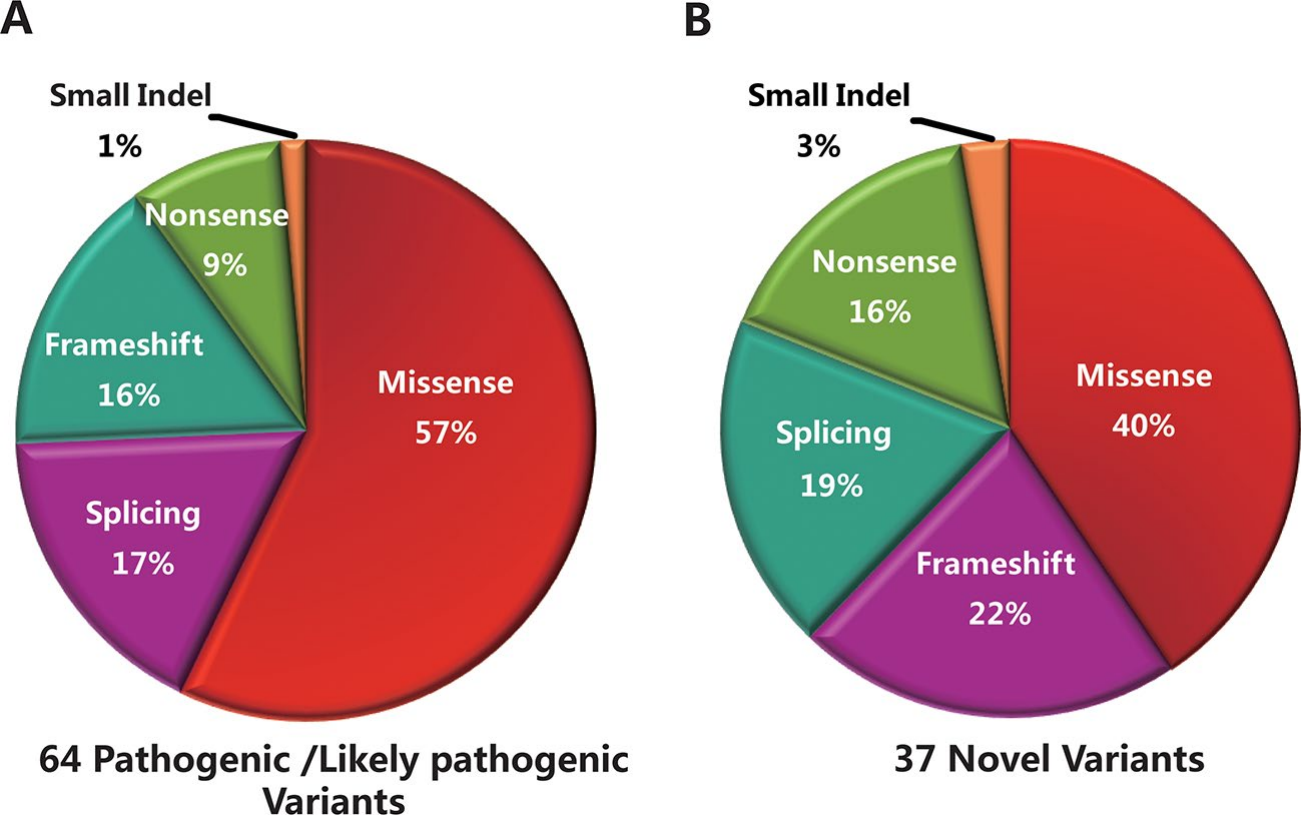
Genetic analyses of the mutant variants identified in the total cohort. **(A)** Sixty-four pathogenic/likely pathogenic variants were identified in this study, including missense (n = 34), splicing (n = 12), frameshift (n = 11), nonsense (n = 6), and small deletion (n = 1) variants. **(B)** Thirty-seven novel variants were identified, including missense (n = 15), frameshift (n = 8), splicing (n = 7), nonsense (n = 6), and small insertion (n = 1) variants.

The 96 mutant variants identified in the total cohort included missense (62/96, 64%), nonsense (6/96, 6%), splicing (15/96, 16%), frameshift (11/96, 12%), and small insertion or deletion (2/96, 2%) variants. Overall, there were 38 pathogenic variants (39.5%), 26 likely pathogenic variants (27.1%), and 32 uncertain-significance variants (33.4%). The majority of uncertain-significance variants were novel variants. The 64 pathogenic/likely pathogenic variants included missense (34/64, 53%), splicing (12/64, 19%), frameshift (11/64, 17%), nonsense (6/64, 9%), and small deletion (1/64, 2%) variants (**Figure 3A**). As shown in **Table 3**, there were 10 prevalent variants in the total cohort. All of these were pathogenic variants, mainly missense variants. The three most prevalent variants were c.101_106delCTTTAT p.Ser34_Leu35del (with an allele frequency of 10.5%), c.2894A > G p.Asn965Ser (6.5%), and c.6563T > C p.Phe2188Ser (4.6%). In addition, the most prevalent variant c.101_106delCTTTAT was found in 16 subjects (15 heterozygous, 1 homozygous). The second most prevalent variant c.2894A > G was found in 10 subjects (all heterozygous). The third most prevalent variant c.6563T > C was detected in 7 subjects (all heterozygous).

**Table 3:** Ten prevalent variants of *ABCA4* gene in 153 subjects.

Gene	Nucleotide change	Amino acid change	Clinical significance^1^	Allele frequency
*ABCA4*	c.101_106 delCTTTAT	p.(Ser34_Leu35del)	Pathogenic	10.5%
*ABCA4*	c.2894A>G	p.(Asn965Ser)	Pathogenic	6.5%
*ABCA4*	c.6563T>C	p.(Phe2188Ser)	Pathogenic	4.6%
*ABCA4*	c.1819G>A	p.(Gly607Arg)	Pathogenic	3.3%
*ABCA4*	c.1006delT^2^	p.(Ser336Profs*38)	Pathogenic	3.3%
*ABCA4*	c.5761G>A	p.(Val1921Met)	Pathogenic	2.6%
*ABCA4*	c.1804C>T	p.(Arg602Trp)	Pathogenic	2.6%
*ABCA4*	c.6282+1G>A	p.?	Pathogenic	2.6%
*ABCA4*	c.6389T>A	p.(Met2130Lys)	Pathogenic	2.6%
*ABCA4*	c.1561delG	p.(Val521Serfs*47)	Pathogenic	2.6%

^1^ABCA4 variants were classified as pathogenic according to the American College of Medical Genetics (ACMG) and genomics guidelines for the more recent cases.

^2^Variant c.1006delT was a novel variant detected in this study.

### Novel Variants of the *ABCA4* Gene

Thirty-seven novel variants were identified in this study (**Supplementary Table S2**), including missense (15/37, 40%), frameshift (8/37, 22%), splicing (7/37, 19%), nonsense (6/37, 16%), and small insertion (1/37, 3%) variants (**Figure 3B**). These novel variants were found in 61 subjects, of whom 56 were in a heterozygous state and 5 homozygous. Of the 37 novel variants, there were 18 pathogenic/likely pathogenic variants (48.6%) and 19 uncertain-significance variants (51.4%). The 18 novel pathogenic/likely pathogenic variants included frameshift (8/18, 45%), nonsense (6/18, 33%), and splicing (4/18, 22%) variants. The uncertain-significance variants mainly included missense variants. Among the 37 novel variants, the most prevalent variant was a frameshift variant c.1006delT with an allele frequency of 3.3% (**Table 3**), which was found in 5 subjects (all heterozygous). Variants c.3055A > G, c.2894A > G, and c.1372C > T were detected in compound heterozygosity with variant c.1006delT. In addition, four variants were detected three times in the cohort, namely, c.6625C > T, c.3634G > T, c.1382_1383delAA, and c.1372C > T. Two frameshift variants c.1006delT and c.1382_1383delAA and a nonsense variant c.6625C > T were identified as pathogenic variants. Furthermore, one novel heterozygous variant c.4561delA was considered as a *de novo* variant in one patient with STGD1, which was not found in either of the parents. Variant c.4561delA was detected in compound heterozygosity with variant c.95C > T.

## Discussion

In the present study, 96 disease-associated *ABCA4* variants were identified in the STGD1 cohort by panel-based NGS technology. Among them, 37 novel variants were detected.

In 153 subjects, the overall variant detection rate for *ABCA4* was 84.3% (129/153), which was not significantly different from the mutation detection rate (70%–90%) in previous studies (Fujinami et al., 2013; Riveiro-Alvarez et al., 2013; Zernant et al., 2014b; Jiang et al., 2016; Smaragda et al., 2018). In recent studies, the variant detection rate for *ABCA4* in STGD1 patients from European and American countries is basically around 80%, while the variant detection rate in the Chinese population can reach more than 90%. In this study, 15 probands were negative after genetic analysis of 792 genes associated with inherited eye diseases. On the one hand, these probands may have large deletion or duplication variants; on the other hand, new pathogenic genes responsible for STGD1 may exist. Therefore, analysis of copy number variants, array CGH, and WGS will be performed on these patients in order to find such variants and important new genes. In addition, similar to previous studies, the vast majority of patients with STGD1 were compound heterozygotes, and only 9 patients were identified as homozygotes. **Supplementary Table S4** lists homozygous variants in STGD1 cases with their respective ages at onset. In this way, the severity of missense variants can be deduced.

Consistent with previous studies (Hargitai et al., 2005; Jiang et al., 2016), 96 *ABCA4* variants identified in this study were distributed throughout the coding region of *ABCA4*. Besides, the variants were all mainly distributed in the TMD1, NBD1, and NBD2 domains of the ABCA4 protein. This suggests that the three functional domains may be high-risk regions in the Chinese cohort, although previous studies of other ethnicities show no variant hot spots in *ABCA4* (Fumagalli et al., 2001; Fujinami et al., 2019).

In accordance with ACMG and genomics guidelines, 64 pathogenic/likely pathogenic variants were identified in this study, of which missense variants accounted for 53%. Besides, 32 uncertain-significance variants were found in 49 subjects, including 28 missense variants, 3 splicing variants, and one small insertion variant. These uncertain-significance variants did not cause significant protein damage, or had an uncertain impact.

In the present study, the three most prevalent variants were c.101_106delCTTTAT (10.5%), c.2894A > G (6.5%), and c.6563T > C (4.6%), which differed from the common variants (c.2424C > G 4.7%, c.6563T > C 3.7%, c.2894A > G 3.1%, and c.101_106delCTTTAT 3.1%) previously reported in a Chinese cohort (Jiang et al., 2016). Moreover, the frequency of common variants in this study was significantly higher than that previously reported in Chinese patients. The most prevalent variant c.101_106delCTTTAT in our study was the third most common variant in the previous Chinese cohort, while the most prevalent variant c.2424C > G previously reported in Chinese patients was only detected in two patients in this study. Based on our subjects, mainly coming from eastern China, it is possible that these three prevalent variants are specific to STGD1 patients in eastern China. Compared with other ethnic groups, the most prevalent variant c.2894A > G in Danish patients (Rosenberg et al., 2007) was the second most common variant in our study. Many common mutant variants identified in other countries were all absent in the present study, such as p.G863A/p.G863del in the Northern European population (Maugeri et al., 1999), p.[L541P;A1038V] in Germany (Rivera et al., 2000), p.R1129L in Spain (Valverde et al., 2006), and p.A1773V in Mexico (Chacon-Camacho et al., 2013). It has been reported that variant c.2894A > G can affect substrate transport across membranes by reducing ATPase activities of the ABCA4 protein (Quazi and Molday, 2013). Based on the analyses of MutationTaster and ABCA4 protein structure, variant c.101_106delCTTTAT in the transmembrane domain might destroy transmembrane α-helices, resulting in the dysfunction of ABCA4 transport. In addition, variant c.6563T > C in the cytoplasmic soluble-protein region responsible for ATP hydrolysis might affect ATPase activities.

Although more than 1,000 variants of *ABCA4* have been reported according to HGMD, recent studies have revealed that there are still novel disease-related variants to be found in STGD1 patients. In the present study, 37 novel variants were identified, including fifteen missense, eight frameshift, seven splicing, six nonsense, and one small insertion variant. The results showed that 37 novel variants were distributed in 19 exons and 7 introns of *ABCA4*. Among them, three different mutant variants were detected in exon 36, and two different variants each in exons 2, 8, 11, 20–22, 25, and 48. Of the 37 novel variants, 18 pathogenic/likely pathogenic variants and 19 uncertain-significance variants were identified according to ACMG and genomics guidelines. The 19 uncertain-significance variants included 16 missense variants with no significant protein damage and 3 splicing variants with no significant impact on splicing. In addition, more than half of the novel variants were detected only once. A frameshift variant c.1006delT was the most prevalent variant among them, which was found in one sporadic case and two families (two probands and two family members). Notably, we identified one *de novo* frameshift variant in the *ABCA4* gene that has not previously been reported, c.4561delA (p.Ile1521Phefs*5), in one patient. This patient presented with an early age of onset and severe clinical symptoms with extensive chorioretinal and RPE atrophy.

## Conclusion

In conclusion, we report here the mutation spectrum of *ABCA4* in a large Chinese cohort, with a total variant detection rate of 84.3%. Our analysis has identified 37 novel disease-associated variants, extending the mutation spectrum of *ABCA4*. Among these, one novel heterozygous variant, c.4561delA, was determined as a *de novo* variant. Moreover, the three most prevalent variants were identified in STGD1 patients from eastern China. Genetic testing of STGD1 patients will improve the accuracy of clinical diagnosis, and improvement of the *ABCA4* mutation spectrum in the Chinese population will be more conducive to gene screening of STGD1 patients in the future.

## Data availability

Our data have been uploaded in the ABCA4 LOVD (www.lovd.nl/ABCA4), which is an almost complete variant and case registry for ABCA4/STGD1.Here is the link: https://databases.lovd.nl/shared/screenings?search_owned_by_=%3D%22Jiankang%20Li%22

## Ethics statement

The study was performed according to the Declaration of Helsinki and approved by the Ethics Committee of the Eye and ENT Hospital of Fudan University. Written informed consent was obtained from all the subjects or their guardians to participate in this study and for the publication.

## Author Contributions

J-HW, G-ZX, S-HZ, and F-YH conceived and designed this study. J-HW, MW, Y-JZ, QC, and G-ZX recruited patients and performed clinical examinations and interpretation. F-JG, F-YH, Y-HQ, PX, D-DW, and S-MS collected the clinical samples and clinical data. F-JG, F-YH, J-KL, L-SW, FC, WL, and S-MS analyzed the sequencing data. J-HW, F-YH, G-ZX, S-HZ, and S-MS wrote and revised the manuscript. The data that support the findings of this study have been deposited in the CNSA (https://db.cngb.org/cnsa/) of CNGBdb with accession code CNP0000503.

## Funding

This work was supported by the National Natural Science Foundation of China (grant NSFC81790641, NSFC81770944, NSFC81870670), the Shanghai Committee of Science and Technology (No.18411965100), the Non-profit Central Research Institute Fund of Chinese Academy of Medical Sciences (2018PT32019), Natural Science Foundation of Guangdong Province (NO.2015A030313472), Shenzhen Engineering Laboratory for Birth Defects Screening (DRC-SZ [2016]750), Shenzhen Municipal Government of China (JCYJ20170412153136375), and a grant from the Research Grants Council of the Hong Kong Special Administrative Region, China (CityU 11256116), and NSFC 61373048.

## Conflict of Interest Statement

The authors declare that the research was conducted in the absence of any commercial or financial relationships that could be construed as a potential conflict of interest.
